# TLR4-Activated MAPK-IL-6 Axis Regulates Vascular Smooth Muscle Cell Function

**DOI:** 10.3390/ijms17091394

**Published:** 2016-08-24

**Authors:** Guan-Lin Lee, Jing-Yiing Wu, Chien-Sung Tsai, Chih-Yuan Lin, Yi-Ting Tsai, Chin-Sheng Lin, Yi-Fu Wang, Shaw-Fang Yet, Yu-Juei Hsu, Cheng-Chin Kuo

**Affiliations:** 1 Institute of Cellular and System Medicine, National Health Research Institutes, Zhunan 35053, Taiwan; lgl0311@gmail.com (G.-L.L.); jywu@nhri.org.tw (J.-Y.W.); ariesw@nhri.org.tw (Y.-F.W.); syet@nhri.org.tw (S.-F.Y.); 2 Graduate Institutes of Life Sciences, National Defense Medical Center, Neihu, Taipei 11490, Taiwan; 3 Division of Cardiovascular Surgery, Department of Surgery, Tri-Service General Hospital, National Defense Medical Center, Neihu, Taipei 11490, Taiwan; sung1500@ndmctsgh.edu.tw (C.-S.T.); linrock@ms26.hinet.net (C.-Y.L.); cvsallen@ndmctsgh.edu.tw (Y.-T.T.); 4 Division of Cardiology, Department of Medicine, Tri-Service General Hospital, National Defense Medical Center, Neihu, Taipei 11490, Taiwan; littlelincs@gmail.com; 5 Institute of Molecular Medicine, National Tsing Hua University, Hsinchu 30013, Taiwan; 6 Division of Nephrology, Department of Medicine, Tri-Service General Hospital, National Defense Medical Center, Neihu, Taipei 11490, Taiwan; yujuei@mail2000.com.tw; 7 Department of Biochemistry, National Defense Medical Center, Neihu, Taipei 11490, Taiwan; 8 Graduate Institute of Basic Medical Science, China Medical University, Taichung 40402, Taiwan

**Keywords:** Toll-like receptor 4, vascular smooth muscle cells, cAMP response element binding protein, Interleukin 6, migration

## Abstract

Migration of vascular smooth muscle cells (VSMCs) into the intima is considered to be a vital event in the pathophysiology of atherosclerosis. Despite substantial evidence supporting the pathogenic role of Toll-like receptor 4 (TLR4) in the progression of atherogenesis, its function in the regulation of VSMC migration remains unclear. The goal of the present study was to elucidate the mechanism by which TLR4 regulates VSMC migration. Inhibitor experiments revealed that TLR4-induced IL-6 secretion and VSMC migration were mediated via the concerted actions of MyD88 and TRIF on the activation of p38 MAPK and ERK1/2 signaling. Neutralizing anti-IL-6 antibodies abrogated TLR4-driven VSMC migration and F-actin polymerization. Blockade of p38 MAPK or ERK1/2 signaling cascade inhibited TLR4 agonist-mediated activation of cAMP response element binding protein (CREB). Moreover, siRNA-mediated suppression of CREB production repressed TLR4-induced IL-6 production and VSMC migration. Rac-1 inhibitor suppressed TLR4-driven VSMC migration but not IL-6 production. Importantly, the serum level of IL-6 and TLR4 endogenous ligand HMGB1 was significantly higher in patients with coronary artery diseases (CAD) than in healthy subjects. Serum HMGB1 level was positively correlated with serum IL-6 level in CAD patients. The expression of both HMGB1 and IL-6 was clearly detected in the atherosclerotic tissue of the CAD patients. Additionally, there was a positive association between p-CREB and HMGB1 in mouse atherosclerotic tissue. Based on our findings, we concluded that, upon ligand binding, TLR4 activates p38 MAPK and ERK1/2 signaling through MyD88 and TRIF in VSMCs. These signaling pathways subsequently coordinate an additive augmentation of CREB-driven IL-6 production, which in turn triggers Rac-1-mediated actin cytoskeleton to promote VSMC migration.

## 1. Introduction

Inflammation is now considered a fundamental process in many important human diseases and health issues such as atherosclerosis [1]. Abnormal activation of components of the innate immune system, such as Toll-like receptors (TLRs), and the consequential chronic inflammation is associated with the development and progression of metabolic inflammatory diseases including atherosclerosis [1]. TLRs, which are pattern recognition receptors, are thought to play a critical role in regulating inflammatory response and in maintaining inflammation homeostasis [2]. Furthermore, inappropriate activation of TLR signaling by pathogenic components and endogenous harmful molecules is thought to be one mechanism initiating and driving inflammatory diseases such as atherosclerosis [3]. Interestingly, TLRs such as TLR1, -2, and -4 have been detected in human atherosclerotic plaques. Importantly, endogenous TLR4 ligands are present in lesions of ApoE-knockout mice and human coronary bypass grafts [4,5], implicating TLR expressions in atherosclerosis. Furthermore, lipopolysaccharide (LPS) activates TLR4-mediated NF-κB and C/EBPβ signaling, leading to increases of pro-inflammatory mediators and subsequent endothelial and vascular damage [4,6]. Absence of TLR4 in mice attenuates atherosclerosis, implicating a role of TLR4 in the pathogenesis of atherosclerotic lesion formation [4,5]. Interestingly, endogenous molecules such as high mobility group box 1 (HMGB1) can activate TLR4, which serves as a late mediator of inflammation and contribute to the development of atherosclerosis [7]. The presence of a loss-of-function TLR4 Asp299Gly polymorphism in human subjects has been reported to be associated with a reduced risk of atherosclerosis [8]. These results further emphasize the importance of TLR4 in promoting atherosclerosis. However, the molecular mechanisms involved in this process remain to be investigated.

It is now well accepted that atherosclerosis is a chronic inflammatory disease. Initial injury to endothelium leads to immune cell infiltration into the vessel wall with subsequent lipid deposition and inflammatory responses [1,9,10], which in turn render medial vascular smooth muscle cells (VSMCs) migratory and proliferative, and secrete pro-inflammatory cytokines and extracellular matrix; these events contribute to development of atherosclerosis [9,10]. Under normal conditions, quiescent VSMCs normally residing in the media of normal blood vessels assume a contractile phenotype [11,12]. Upon endothelial injury signals, VSMCs migrate into the intima and undergo a high proliferative and synthetic phenotypic switch [13]. Furthermore, they release considerable pro-inflammatory cytokines such as interleukin 6 (IL-6) to result in vascular inflammation [14]. Given the role of TLR4 in inflammation and in regulating VSMC phenotypic modulation, it is important to uncover the precise mechanism by which TLR4 regulates VSMC function and atherosclerosis.

The transcription factor CREB (cAMP response element-binding protein) plays diverse roles in regulating cellular functions including cell proliferation, survival, and differentiation. Many growth factors and inflammatory signals induce CREB activation and expression, which in turn mediate the transcription of diverse genes harboring cAMP-response elements [15,16]. Typically, CREB is activated by protein kinases-mediated phosphorylation at serine 133, which allows binding to the co-activator protein, CREB-binding protein (CBP), and drives CREB-mediated gene expression of genes including pro-inflammatory IL-6 [15,17]. Further, TLRs-induced CREB-mediated IL-6 transcription is mediated by triggering CREB Ser-133 phosphorylation in immune cells [17]. Indeed, CREB has been reported to promote VSMC migration and proliferation [18,19,20], but the underlying triggering mechanisms are still unclear. We hypothesized that TLR4 regulates VSMC migration through the activation of CREB-mediated IL-6 production. Therefore, in this study, we evaluated the effect of TLR4 agonists including LPS and HMGB1 on VSMC migration.

## 2. Results

### 2.1. LPS/TLR4 Induced Production of IL-6, But Not IL-10, IL-12, and TNF-α, in VSMCs

**Figure 1:**
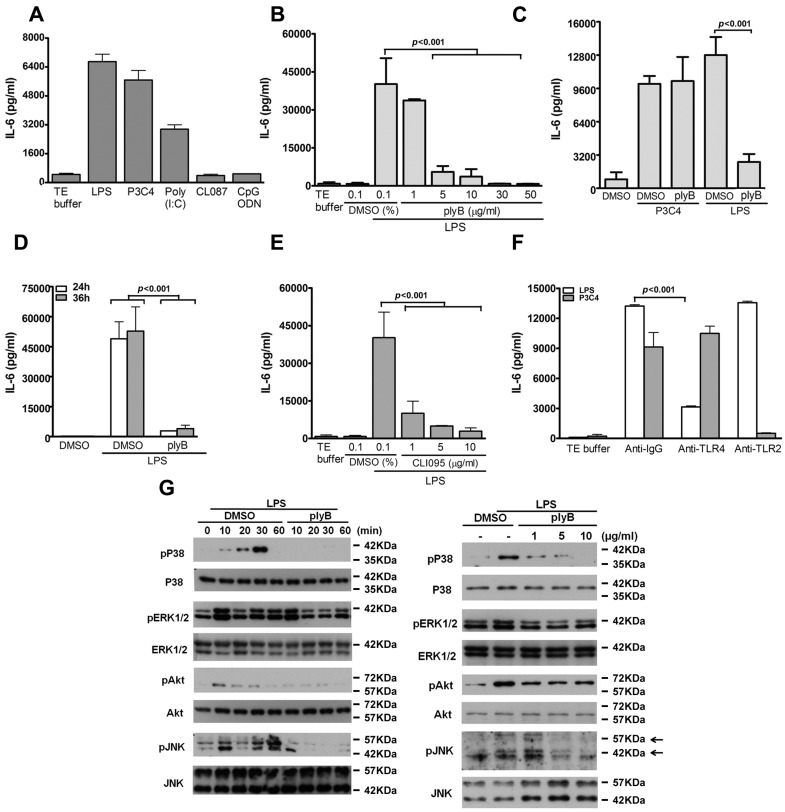
Activation of TLR4 signaling in vascular smooth muscle cells (VSMCs) induces IL-6 production. (**A**–**F**) IL-6 level in culture medium was measured using enzyme-linked immunosorbent assay (ELISA); (**A**) Serum-starved VSMCs were stimulated with various TLR ligands: 100 ng/mL lipopolysaccharide (LPS) (TLR4), 1 μg/mL pam3CSK4 (TLR2), 1 μg/mL Poly (I:C) (TLR3), 1 μg/mL CL087 (TLR7), or 1 μM CpG ODN (TLR9) for 24 h. After pretreating VSMCs with different concentrations of (**B**–**D**) polymyxin B (plyB; LPS inhibitor) or (**E**) CLI-095 (TLR4 inhibitor) for 30 min, the cells were stimulated with LPS (100 ng/mL) for 24 h (**B**–**E**) or 36 h (**D**); *p* < 0.001 for LPS + DMSO vs. LPS + ply or LPS + CLI-095. (**F**) LPS-treated VSMCs were further treated with anti-IgG, antibodies (5 μg/mL) against TLR2 or TLR4 for 24 h. *p* < 0.001 vs. anti-IgG. Data in **A**–**F** represent mean ± SD of three experiments. Statistical analyses were performed using the one-way analysis of variance (ANOVA); (**G**) VSMCs were pretreated with 5 μg/mL plyB or with different amounts of plyB for 30 min and then stimulated with TE buffer or LPS for the indicated times (**left panel**) or for 30 min (**right panel**). Cell lysates were subjected to Western blotting with antibodies against p38 mitogen-activated protein kinase (MAPK), phospho-p38 MAPK, ERK1/2, phospho-ERK1/2, Akt, phospho-Akt, JNK1/2, phospho-JNK1/2, or β-actin. A representative of three independent experiments is shown.

To investigate the role of TLR4 in the production of pro-inflammatory cytokines by VSMCs, quiesced VSMCs were stimulated with the TLR4 ligand, LPS, for 24 h, and the levels of pro-inflammatory cytokines (IL-6, IL-10, IL-12, and TNF-α) in the culture medium were measured. LPS substantially stimulated IL-6 production (Figure 1A) but did not stimulate the production of TNF-α, IL-10 or IL-12, (data not shown). As with LPS, the ligands of TLR2 (pam3CSK4) and TLR3 (Poly(I:C)) significantly induced IL-6 generation, while the agonists for TLR7/8 (CL087) and TLR9 (CpG ODN) did not (Figure 1A).

To determine the specificity of LPS action on IL-6 production, VSMCs were treated with a specific LPS inhibitor, polymyxin B , which has been reported to form a stable complex with the lipid A moiety of LPS [21]. Polymyxin B dose-dependently abolished the LPS-induced IL-6 production, but not the pam3CSK4-induced IL-6 production (Figure 1B,C). In addition, LPS-driven IL-6 production was suppressed by polymyxin B (5 μg/mL) at 24 and 36 h time points (Figure 1C,D).

We next evaluated the essential role of TLR4 in IL-6 production by LPS-stimulated VSMCs. VSMCs were treated with another specific TLR4 inhibitor, CLI-095, which specifically suppresses TLR4 signaling by blocking the intracellular domain of TLR4 [22]. The results revealed that CLI-095 dose-dependently suppressed LPS-induced IL-6 production (Figure 1E), but not pam3CSK4-induced IL-6 production (data not shown). Furthermore, the increase in IL-6 production by the VSMCs was abolished by anti-TLR4 but not by control anti-IgG and anti-TLR2 antibodies (Figure 1F); however, IL-6 production induced by pam3CSK4 was unaffected by anti-TLR4 antibodies, but treatment with anti-TLR2 antibodies suppressed the IL-6 production (Figure 1F). These results suggest that TLR4 participates in LPS-induced IL-6 production in VSMCs.

LPS-mediated activation of immune cells is through the TLR4 signaling cascade, which implicates multiple kinases including p38 MAPK, ERK1/2, JNK1/2, and phosphatidylinositol 3′-kinase (PI3K) [23]. We therefore next determined whether LPS activates the TLR4-dependent kinase signaling pathway in VSMCs in a manner similar to that in immune cells. We found that LPS treatment induced a strong phosphorylation of p38 MAPK, ERK1/2, AKT, and JNK1/2 within 10 min, which lasted for at least 30 min; this induced phosphorylation was suppressed by treatment with polymyxin B in a time- and dose-dependent manner (Figure 1G). Taken together, these results suggest that the TLR4 signaling cascade activated by LPS in mouse VSMCs is similar to that in mouse immune cells, which has been previously reported [2,24].

### 2.2. Role of TLR4 in LPS-Induced VSMC Migration

**Figure 2:**
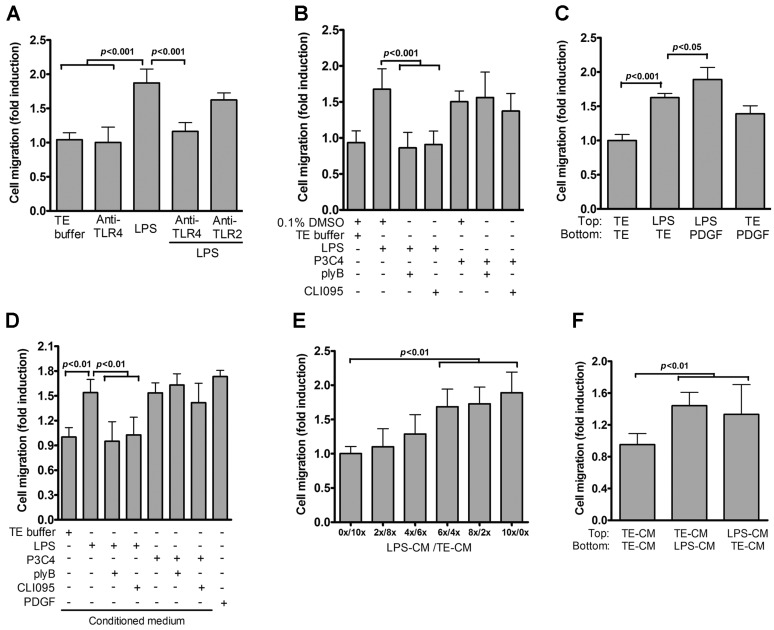
Role of TLR4 in VSMC migration. (**A**) VSMCs (in the presence or absence of anti-TLR2 or anti-TLR4 antibodies (5 μg/mL)) were treated with TE buffer or LPS for 24 h; (**B**) Quiesced VSMCs were stimulated with TE buffer, LPS or P3C4 in the presence or absence of plyB or CLI-095 for 24 h. Migration assays were then performed with PDGF-BB as a chemoattractant. *p* < 0.001 vs. LPS or LPS + DMSO; (**C**) Serum-starved VSMCs were seeded into the upper chambers of transwell plates and stimulated with TE buffer or LPS for 24 h. Migration assays were then performed with or without PDGF-BB as a chemoattractant. *p* < 0.001 for LPS vs. TE. *p* < 0.05 for LPS vs. LPS + PDGF; (**D**) Conditioned medium from VSMCs stimulated with TE buffer, LPS or P3C4 with or without plyB or CLI-095 for 24 h was collected and added to the lower chamber as a chemoattractant in the transwell migration assays. PDGF-BB was used as a positive control. *p* < 0.01 vs. LPS; (**E**) Serial dilutions of conditioned medium (CM) from VSMCs stimulated with LPS (LPS-CM) were prepared by diluting with TE-treated CM (TE-CM) and added to the lower chambers; VSMCs were plated onto top chambers of transwells. After 24 h incubation, VSMC migration was determined by the transwell assay. *p* < 0.01 vs. 0x/10x; (**F**) Checkerboard assays. CM from VSMCs treated with TE buffer (TE-CM) or LPS (LPS-CM) for 24 h was added either to the upper or lower chamber of the transwell plates for VSMC migration assays. *p* < 0.01 vs. TE-CM/TE-CM. Data in **A**–**F** represent mean ± SD of three experiments. Statistical analyses were performed using the one-way ANOVA.

VSMC migration is a key event in atherosclerosis progression [9,10]. We wondered if LPS stimulates VSMC migration. LPS markedly increased VSMC migration as compared with that observed with endotoxin-free TE buffer (Figure 2A). We next used the anti-TLR4 neutralization assay to characterize the potential role of TLR4 in LPS-induced VSMC migration. Anti-TLR4, but not anti-TLR2 antibodies, reduced LPS-induced VSMC migration (Figure 2A). Additionally, LPS-mediated VSMC migration was suppressed by treatment with polymyxin B and CLI-095, while pam3CSK4-induced VSMC migration was unaffected by the two inhibitors (Figure 2B), suggesting that TLR4 is required for VSMC migration induced by LPS. Next, to gain mechanistic insights of LPS-induced migration, we used TE buffer or PDGF-BB as chemoattractant to assay for random and directed migration, respectively. The directed migration of TE-treated VSMCs was induced by PDGF-BB as a chemoattractant placed in the bottom chambers (Figure 2C), indicating that VSMCs can respond to chemoattractant stimulation. When TE buffer was placed in the bottom chambers, the random migration of LPS-treated VSMCs was 1.6-fold that of TE-treated cells (Figure 2C), while PDGF-BB placed in the bottom chambers further enhanced the migration of VSMCs treated with LPS (Figure 2C). Taken together, these results indicate that the enriched migration of VSMCs following LPS stimulation was due to augmented random and directed migration.

We wondered if TLR4-mediated VSMC migration could be attributable to the secretion of soluble pro-migratory molecules. To address this, the conditioned medium (CM) from LPS-treated VSMCs, rather than that from PDGF-BB-treated VSMCs, was used as a chemoattractant in transwell migration assays. CM from LPS-treated VSMCs (LPS-CM) significantly augmented VSMC migration (Figure 2D) as compared with that observed with the control CM (TE-CM). Further, pretreatment with polymyxin B or CLI-095 did not affect the migration mediated by LPS-CM, indicating that the ability of LPS-CM to promote VSMC migration was not due to LPS contamination (data not shown). The extent of VSMC migration induced by LPS-CM was comparable with that induced by PDGF (Figure 2D). However, CM from VSMCs co-stimulated with LPS and either polymyxin B or CLI-095 could not induce VSMC migration (Figure 2D).

To assess the involvement of soluble chemotactic factors of the LPS-CM in indicating VSMC migration, we prepared serial LPS-CM dilutions and used them as a chemoattractant in the transwell assay system. VSMC migration enhanced as the concentration of LPS-CM in the dilutions decreased (Figure 2E). Taken together, these results indicate that LPS-stimulated VSMCs release soluble factors to induce VSMC migration. Next, we performed checkerboard analysis to confirm that the effect of LPS-CM on inducing VSMC migration was due to either chemotaxis or chemokinesis (Figure 2F). VSMC migration assays were performed by placing LPS-CM in the upper or bottom chambers. VSMC migration was significantly enhanced regardless of where LPS-CM was placed, suggesting that LPS-CM could induce both chemotactic (directed) and chemokinetic (nondirected) migration.

### 2.3. IL-6 Is Essential for TLR4-Induced VSMC Migration

**Figure 3:**
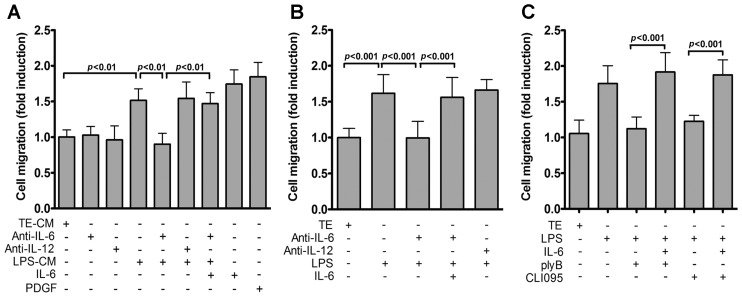
IL-6 mediates TLR4 signaling-induced VSMC migration. (**A**) VSMCs were treated with TE buffer or LPS and conditioned medium collected. Migration assays were then performed using conditioned medium (with or without anti-IL-6, anti-IL-12 antibodies (5 μg/mL) or recombinant IL-6 (50 ng/mL)) as chemoattractants. Migrated cells were then counted 24 h later. *p* < 0.01 vs. LPS-CM or LPS-CM + anti-IL-6; (**B**) Serum-starved VSMCs were stimulated with TE buffer or LPS with or without anti-IL-6, anti-IL-12 antibodies or IL-6 (50 ng/mL) for 24 h and migration assays performed as in Figure 2A. *p* < 0.001 vs. LPS or LPS + anti-IL-6; (**C**) VSMCs (in the presence or absence of plyB, CLI-095 or IL-6) were treated with TE buffer or LPS for 24 h. VSMC migration was then measured by the transwell assays. *p* < 0.001 vs. LPS + ply or LPS + CLI-095. Data in (**A**–**C**) represent mean ± SD of three experiments. Statistical analyses were performed using the one-way ANOVA.

The involvement of IL-6 in regulating VSMC migration has been reported [19,25]. Given that TLR4 inhibition attenuated IL-6 levels and VSMC migration (Figure 1 and Figure 2), it is likely that IL-6 is released into conditioned medium to function as a soluble factor. To test this, we evaluated whether IL-6-neutralizing antibodies could affect the migration-inducing activity of LPS-CM. LPS-CM-induced VSMC migration was significantly inhibited by IL-6- but not IL-12-neutralizing antibodies. This inhibitory effect of anti-IL-6 antibody was restored by administration of exogenous recombinant mouse IL-6 (Figure 3A). To further confirm this finding, IL-6-neutralizing antibodies were added prior to LPS stimulation. Neutralizing antibodies against IL-6, but not IL-12, abrogated LPS-induced VSMC migration (Figure 3B). Furthermore, exogenous recombinant mouse IL-6 restored LPS-mediated VSMC migration that was inhibited by either polymyxin B or CLI-095 (Figure 3C). Collectively, our data suggest that IL-6 in the extracellular milieu mediates TLR4 activation-induced VSMC migration.

### 2.4. TLR4-Induced IL-6 Production and VSMC Migration Are Mediated by p38 MAPK and ERK1/2

**Figure 4:**
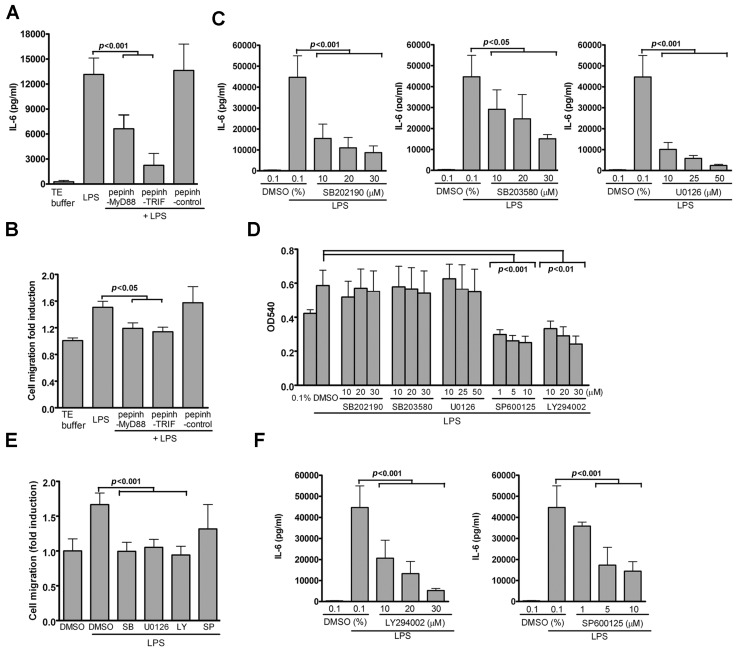
TLR4-mediated IL-6 production and VSMC migration depend on p38 MAPK and ERK1/2 pathway-mediated by Myd88 or TRIF. VSMCs were pretreated with pepinh-MyD88 (Myd88 signaling inhibitor) or pepinh-TRIF (TRIF signaling inhibitor) for 30 min, then stimulated with LPS for 24 h. (**A**) IL-6 levels in culture supernatants were measured by ELISA. *p* < 0.001 vs. LPS; (**B**) VSMC migration was measured by the transwell assays. *p* < 0.05 vs. LPS; (**C**–**F**) VSMCs were pretreated with various concentrations of the indicated inhibitors (SB202190 or SB203580 for p38; U0126 for ERK1/2; SP600125 for JNK; or LY294002 for PI3K) for 30 min and then stimulated with LPS for 24 h; (**C**) IL-6 levels in culture supernatants were measured by ELISA. *p* < 0.001 or 0.05 vs. LPS; (**D**) MTT assay was used to determine cell viability. *p* < 0.001 vs. LPS with SP600125; *p* < 0.01 vs. LPS with LY294002; (**E**) VSMC migration was measured by the transwell assays; (**F**) IL-6 levels in culture supernatants were measured by ELISA. *p* < 0.001 vs. LPS + dimethyl sulfoxide (DMSO). Data in **A**–**F** represent mean ± SD of three experiments. Statistical analyses were performed using the one-way ANOVA.

Since TLR4-mediated cellular effects signal through adaptor protein MyD88 and TRIF [23], we used well-characterized peptide inhibitors to examine the roles of the MyD88 and TRIF pathways in TLR4-induced IL-6 production in the VSMCs. Peptide blockers of MyD88 and TRIF, but not control peptide blockers, significantly suppressed IL-6 production and VSMC migration induced by LPS (Figure 4A,B) without affecting VSMC viability (data not shown), suggesting that both MyD88 and TRIF pathways contribute to LPS/TLR4-mediated cellular effects in VSMCs.

Both MyD88- and TRIF-mediated signaling have been shown to activate several kinases. Furthermore, given that LPS activates p38 MAPK, ERK1/2, and JNK1/2, as well as PI3K/AKT (Figure 1G), we performed pharmacological inhibition to evaluate the effect of these kinases to LPS-induced IL-6 production. Dose-dependent inhibition of LPS-induced IL-6 production in the VSMCs by p38 and ERK1/2 by inhibitors SB202190, SB203580, and U0126 was detected without affecting VSMC viability, respectively (Figure 4C,D). These results indicate that p38 MAPK and ERK1/2 are involved in LPS-induced IL-6 production in VSMCs.

Since IL-6 production was found to play a role in LPS-induced VSMC migration (Figure 3), we investigated whether the above-mentioned two kinases, p38 MAPK or ERK1/2, were also involved in VSMC migration. LPS-induced migration was diminished by p38 MAPK or ERK1/2 inhibitors in a manner similar to that observed for IL-6 production (Figure 4E). Although the PI3K inhibitor LY294002 and the JNK1/2 inhibitor SP600125 inhibited LPS-induced IL-6 production (Figure 4F) and VSMC migration (Figure 4E), the two inhibitors also resulted in VSMC death (Figure 4D), suggesting that the influence of these two inhibitors on LPS-induced cellular effects was due to cellular toxicity. Taken together, these results indicate that TLR4-induced IL-6 production and VSMC migration are mediated by the concerted actions of MyD88 and TRIF on activating p38 MAPK and ERK1/2 signaling.

### 2.5. CREB-Mediated IL-6 Production is Crucial for LPS-Induced VSMC Migration

**Figure 5:**
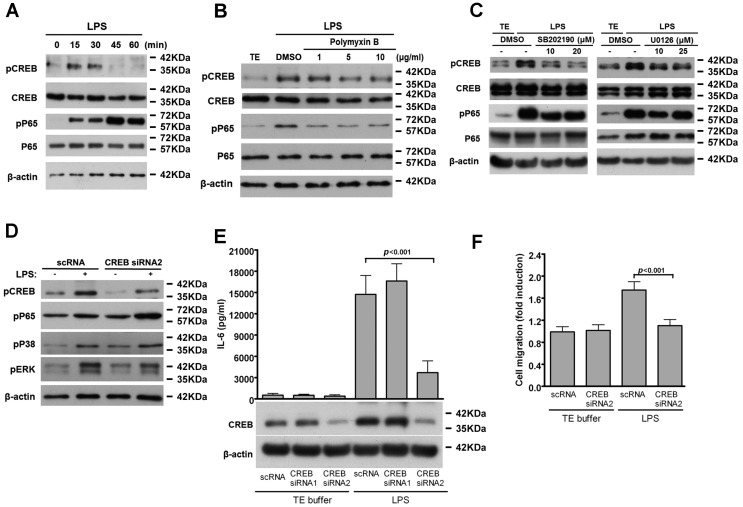
CREB-mediated IL-6 production is involved in LPS-induced VSMC migration. (**A**) Serum-starved VSMCs were stimulated with LPS for the indicated times; (**B**,**C**) VSMCs were pretreated with different amounts of polymyxin B (**B**); SB202190 or U0126 (**C**) for 30 min and then stimulated with LPS for 30 min. Cell lysates were subjected to Western blotting with antibodies for CREB, phospho-CREB, NF-κB p65, phospho-NF-κB p65 (Ser536) or β-actin; (**D**–**F**) VSMCs were transfected with the indicated siRNAs for 24 h and then serum depleted for 24 h; (**D**) Cells were incubated with LPS for 30 min. Cell lysates were immunoblotted with antibodies for CREB, phospho-CREB, phospho-p38, phospho-ERK or β-actin; (**E**) Cells were stimulated with LPS for 24 h and IL-6 level in culture supernatants and CREB level in cell lysates were measured by ELISA and western blot, respectively; (**F**) Quiesced VSMCs were incubated with LPS for 24 h and migration assays performed as in Figure 2A. *p* < 0.001 vs. LPS + scRNA. The experiments in **A**–**D** were repeated three times with similar results. Data in **E**–**F** represent mean ± SD of three experiments. Statistical analyses were performed using the one-way ANOVA.

Several cis-elements and cognate transcription factors such as CREB and NF-κB in the promoter region of IL-6 gene controls IL-6 transcription [26]. Therefore, we wondered whether LPS activated these two transcription factors in VSMCs. In our previous experiment, LPS induced the phosphorylation of CREB and NF-κB p65 (Ser536) in VSMCs within 10 min, which lasted for at least 30 min (Figure 5A), and this induction was abrogated by polymyxin B (Figure 5B). Further, since our results (Figure 4) indicate that both p38 MAPK and ERK1/2 signaling were involved in LPS-induced IL-6 production, we reasoned that these pathways might mediate LPS-induced activation of CREB and NF-κB p65. Interestingly, inhibiting p38 MAPK or ERK1/2 signaling significantly reduced CREB but not NF-κB p65 (Ser536) phosphorylation (Figure 5C), indicating that p38 MAPK and ERK1/2 signaling mediates LPS-stimulated CREB phosphorylation.

CREB has been reported to play a critical role in VSMC migration [18,19,27]; therefore, we used CREB siRNA to determine whether CREB is essential for LPS-induced IL-6 production and VSMC migration. CREB siRNA2 decreased CREB expression and the LPS-induced phosphorylated CREB level in the VSMCs without affecting the phosphorylation level of p65, p38, and ERK1/2 (Figure 5D). In addition, silencing of CREB by CREB siRNA2 resulted in a marked reduction in IL-6 production; this effect was not observed with control scRNA and CREB siRNA1 (Figure 5E). Moreover, LPS-induced VSMC migration was blocked by CREB siRNA2 but not by control scRNA (Figure 5F). These results provide evidence that CREB-mediated IL-6 expression is required for LPS-induced VSMC migration.

### 2.6. Rac1-Mediated Actin Stress Fiber Formation Participates in LPS-Induced VSMC Migration

**Figure 6:**
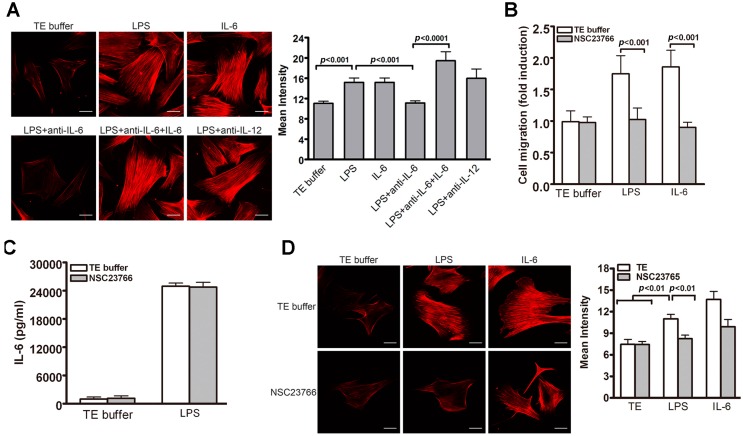
Role of Rac1-mediated F-actin formation in TLR4-induced VSMC migration. (**A**) VSMCs were subjected to different treatments (TE, LPS, IL-6, and in combination with anti-IL-6 or IL-12) for 24 h. Rhodamine-conjugated phalloidin staining was then performed to reveal actin stress fibers of VSMCs. The staining intensity was then quantified. *p* < 0.001 vs. LPS; *p* < 0.0001 vs. LPS + anti-IL6; (**B**,**C**) VSMCs were pretreated with Rac1 inhibitors NSC23766 (100 μM) for 30 min before stimulation with TE buffer, LPS or IL-6 for 24 h; (**B**) Migration assays were performed using transwell assays and PDGF-BB as a chemoattractant. *p* < 0.001 for TE buffer vs. NSC23766; (**C**) ELISA was performed to determine IL-6 levels in culture medium; (**D**) VSMC Phalloidin staining and quantitative analysis of staining intensity. *p* < 0.01 vs. LPS with TE buffer. Representative images of three independent experiments are shown in **A** and **D**. Scale bar, 5 μm. One-way ANOVA was used for statistical analyses in **A**–**D**.

As actin cytoskeleton reorganization plays a key role in regulating cell migration, we thus examined actin polymerization of VSMCs after LPS stimulation. Rhodamine-conjugated phalloidin staining (for filamentous actin fiber (F-actin)) revealed that LPS significantly induced actin stress fiber formation (Figure 6A). Interestingly, IL-6- but not IL-12-neutralizing antibodies suppressed stress fiber formation (Figure 6A). This anti-IL-6 antibody-mediated suppression of LPS-induced F-actin organization in the VSMCs was restored by IL-6 administration (Figure 6A), indicating that LPS-induced F-actin formation in VSMCs is mediated via the release of IL-6 in the conditioned medium.

Rac1, a member of the Rho family of small GTP-binding proteins, regulates cellular migration through actin cytoskeletal structure assembly [28]. To investigate whether Rac1 participates in TLR4-induced VSMC migration, we treated VSMC with NSC23766, an inhibitor of Rac1. NSC23766 significantly reduced VSMC migration induced by LPS and IL-6 (Figure 6B) but did not inhibit LPS-induced IL-6 production (Figure 6C). In addition, LPS- or IL-6-induced actin stress fibers were significantly attenuated by NSC23766 (Figure 6D). Together, our results suggest that Rac1 plays a critical role in LPS-induced VSMC migration by inducing F-actin formation.

### 2.7. IL-6 Level Is Positively Correlated with P-CREB Level in Patients with Coronary Artery Disease (CAD)

**Figure 7:**
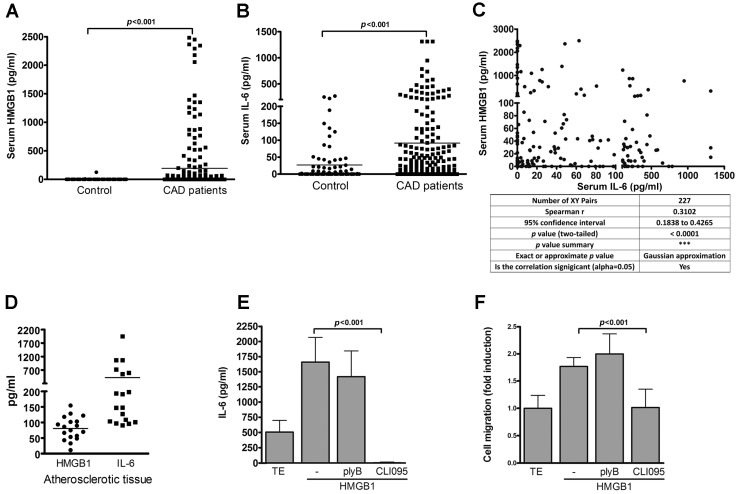
TLR4 endogenous ligand HMGB1 induces IL-6 production and VSMC migration. (**A**) Serum HMGB1 concentrations in CAD patients (*n* = 227) and healthy subjects (*n* = 54). *p* < 0.001 vs. control; (**B**) Serum IL-6 concentrations in CAD patients (*n* = 227) and healthy subjects (*n* = 80) were measured by ELISA. *p* < 0.001 vs. control. Statistical analyses in A-B were performed using the *t*-test; (**C**) Correlation between serum HMGB1 and serum IL-6 in CAD patients (*n* = 227). Spearman’s rank correlation coefficient (*r* = 0.3102, *p* < 0.0001). All values are expressed as means ± SD; (**D**) Amounts of HMGB1 and IL-6 in the atherosclerotic tissue of CAD patients were measured by ELISA; (**E**,**F**) VSMCs were pretreated with plyB or CLI-095 for 30 min, then stimulated with HMGB1 (50 μg/mL) for 24 h; (**E**) IL-6 levels in culture supernatants were measured by ELISA; (**F**) VSMC migration was measured by the transwell assays. *p* < 0.001 vs. HMGB1. Data in **E**,**F** represent mean ± SD of three experiments. Statistical analyses in **E**–**F** were performed using the one-way ANOVA.

Given that TLR4 induced IL-6 production in VSMCs, we investigated the clinical correlation between the TLR4 endogenous ligand, HMGB1, and IL-6 production in human patients with CAD. Serum HMGB1 (192.8 ± 33.24 pg/mL) and IL-6 (91.7 ± 13.5 pg/mL) levels in human CAD patients were significantly higher than those in the control subjects (HGB1: 2.5 ± 33.24 pg/mL; IL-6: 26.7 ± 6.42 pg/mL) (Figure 7A,B). The serum HMGB1 level was also found to be positively correlated with serum IL-6 level in the CAD patients (Figure 7C).

In addition, both HMGB1 (80.6 ± 8.74 pg/mL) and IL-6 (418.8 ± 118.6 pg/mL) were clearly detected in the atherosclerotic tissues of the CAD patients (Figure 7D). Further, our results revealed that, like LPS, HMGB1 induced IL-6 production by the VSMCs and promoted VSMC migration (Figure 7E,F), which were suppressed by treatment with the specific TLR4 signaling inhibitor, CLI-095, but not by treatment with the LPS binding inhibitor, polymyxin B (Figure 7E,F).

**Figure 8:**
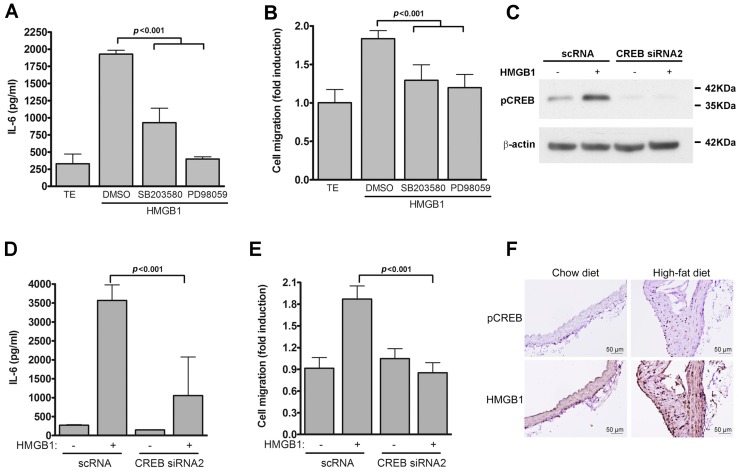
CREB activation is essential for HMGB1-induced IL-6 production and migration of VSMCs. (**A**,**B**) VSMCs were pretreated with different concentrations of the indicated inhibitors for 30 min and then stimulated with HMGB1 for 24 h; (**A**) ELISA was used to measure IL-6 levels in culture supernatants; (**B**) Transwell migration assays were performed to measure VSMC migration. *p* < 0.001 vs. HMGB1; (**C**–**E**) VSMCs were transfected with the indicated siRNAs for 24 h and then serum depleted for 24 h; (**C**) Cells stimulated with HMGB1 for 30 min and cell lysates prepared. Western blot analysis was then performed with antibodies to detect phospho-CREB or β-actin; (**D**) Culture supernatants from cells incubated with HMGB1 for 24 h was prepared and IL-6 levels measured by ELISA. *p* < 0.001 vs. HMGB1 + scRNA; (**E**) Quiesced VSMCs were incubated with HMGB1 for 24 h and migration assays performed as in Figure 2A. *p* < 0.001 vs. HMGB1 + scRNA. Data in **A**–**E** represent mean ± SD of three experiments. Statistical analyses were performed using the one-way ANOVA; (**F**) Immunohistochemistry on a mouse aortic arch stained with antibodies for p-CREB as well as HMGB1 and counterstained with hematoxylins. Scale bar, 50 μm.

Additionally, HMGB1-induced IL-6 production and VSMC migration were suppressed by treatment with the p38 inhibitor, SB203580, and the ERK1/2 inhibitor, PD98059, suggesting that HMGB1-induced IL-6 production and VSMC migration were signaled via the TLR4-driven p38 MAPK- and ERK1/2-dependent pathways (Figure 8A,B). Since CREB activation is required for LPS/TLR4-mediated IL-6 production and VSMC migration, as demonstrated in Figure 5E,F, we next investigated whether CREB activation was essential for HMGB1-induced IL-6 production and VSMC migration. CREB siRNA2 decreased HMGB1-induced phosphorylated CREB level in the VSMCs (Figure 8C), accompanied by inhibition of HMGB1-induced IL-6 production and VSMC migration (Figure 8D,E).

To further determine the physiological or pathological relevance of p-CREB with HMGB1 in mouse atherosclerotic lesions, we next studied the expression and distribution of p-CREB and HMGB in high-fat diet-induced atherosclerotic plaques of apolipoprotein E-deficient (ApoE−/−) mice. Immunohistochemical staining revealed that a significant amount of p-CREB was present in the atherosclerotic lesions of the ApoE−/− mice fed a high-fat diet, but not in the control arteries of the mice fed a standard chow diet; moreover, the presence of p-CREB was mainly restricted to VSMCs within the atherosclerotic neointima (Figure 8F). We also found that HMGB1 was highly expressed in the atherosclerotic lesions, which is consistent with a previous report [29]. Importantly, the protein expression of p-CREB was positively associated with HMGB1 level (Figure 8F). This finding emphasizes that CREB-mediated IL-6 production plays a critical role in TLR4-mediated VSMC migration.

## 3. Discussion

Cardiovascular disease remains a major health issue worldwide, with significant morbidity and mortality. The main underlying cause of cardiovascular disease is atherosclerosis, which is recognized as a chronic inflammatory disease. In response to hypercholesterolemia, low-density lipoprotein (LDL) particles from the blood infiltrate into subendothelial intima space, resulting in LDL retention in the intima, which initiates an inflammatory response in the arterial wall [1]. In the early process of atherosclerosis, arterial cells, particularly endothelial cells and macrophages, proceed to induce the production of lipid peroxides, which leads to foam cell formation and creates fatty streak [1]. Subsequently, the inflammatory microenvironment of the atherosclerotic lesion induces medial SMC proliferation and migration into the intima; however, the full-spectrum of agonists for stimulating VSMCs remains to be determined so far. Despite that TLR4 plays a pathogenic role in atherosclerosis through its actions on endothelial cell activation and macrophage recruitment [4,30], its role in VSMC proliferation and migration and the precise mechanisms are still not well understood. Heat shock protein 60 and 70, fibronectin, oxidized LDL, hyaluronan, and HMGB1, which have been reported to be TLR4 ligands, are detected in the plaques of ApoE-knockout mice and human coronary bypass grafts [1], indicating that these molecules could be a potential endogenous ligands of TLR4. In this study, we further demonstrated that TLR4 activation mediates VSMC migration through an IL-6-driven signaling pathway. Ligand-activated TLR4 primarily drives CREB transactivation to induce IL-6 expression at the transcription level, and subsequent IL-6 secretion into the extracellular milieu, which in turn promotes VSMC migration through Rac1-driven F-actin polymerization. Our findings not only demonstrate that TLR4 signals via mitogen-activated protein kinases (MAPK) including p38 MAPK and ERK1/2 that are coordinately regulated by MyD88 and TRIF, but also provide novel evidence that these signaling pathways are involved in the TLR4-triggered CREB-mediated IL-6 production and VSMC migration. Our results from the in vitro experiments conducted using primary VSMCs provide new insights into the mechanism underlying TLR4-induced VSMC migration. However, to further confirm the role of TLR4 ligand-induced IL-6 production in the pathogenesis of atherosclerosis, in vivo studies using TLR4-deficient mice should be conducted in the future.

The homeostasis in the crosstalk between endothelial cells (ECs) and VSMCs has been considered a key factor in vascular homeostasis. Recent reports have indicated that EC-derived microRNAs not only maintain EC homeostasis but also control VSMC functions including proliferation and migration [31,32]. In this study, although we have provided new mechanistic insights into TLR4-induced VSMC migration, the connection between EC-derived microRNAs and TLR4-mediated VSMC migratory signaling remains to be investigated.

In the innate immune system, TLRs are principal receptors providing a mechanistic link between vascular inflammation and diseases such as atherosclerosis. Individual TLRs exhibit specific responses by interacting with a variety of adapter proteins and activate many different transcription factors; therefore, the dissection of individual TLR signaling in VSMC function may provide insights into treatments for atherosclerosis. Several TLRs have been found to be expressed in VSMCs, among which only three, TLR2, TLR3, and TLR4, participate in the induction of IL-6 production by VSMCs. Our previous report indicated that TLR2 signaling mediates VSMC migration but not proliferation [20]. In comparison, TLR4 signaling induces both VSMC migration and proliferation [33,34]. Further, previously, inhibition of the PI3K/AKT signaling pathway was reported to suppress HMGB1-induced VSMC migration [34]; however, in the present study, we found that the PI3K inhibitor suppressed HMGB1-induced VSMC migration, which was accompanied by decreased cell viability. In contrast, inhibitors of ERK1/2 and p38 indeed suppressed TLR4-mediated VSMC migration without affecting cell viability, which suggested that ERK1/2 and p38, but not PI3K, are directly attributed to TLR4-mediated VSMC migration. Further, we demonstrated that activation of TLR4 by LPS and HMGB1 triggers p38 MAPK and ERK1/2 signaling in VSMCs. We demonstrate that these two signaling pathways converge to activate CREB. CREB activation induces IL-6 transcription and production, leading to Rac-1 activation, actin cytoskeleton organization, and ultimately increased VSMC migration. It is of interest to note that TLR3 signaling did not affect VSMC migration (our unpublished data). However, TLR3 signaling has been reported to render human VSMCs toward a pro-inflammatory and proliferative phenotype [35]. Furthermore, deficiency of TLR3 in hypercholesterolemic ApoE−/− mice dramatically increased atherogenic lesion, suggesting that TLR3 has a protective function in the arterial wall [36]. Taken together, these data propose that different TLRs might play distinct roles in VSMCs and vascular diseases.

IL-6 is a pleiotropic cytokine and has been implicated in chronic inflammation and atherogenesis. Upon PDGF-BB, thrombin, angiotensin II, and 15(*S*)-hydroxyeicosatetraenoic acid stimulation, VSMC increases IL-6 production. Further, IL-6 stimulation has been reported to play a key role in inducing VSMC migration and proliferation [19,25,37,38]. Our results from the present study reveal that TLR4 ligands, LPS or HMGB1, induce the production of IL-6 but not that of other cytokines including IL-12, IL-10, and TNF-α in VSMCs. IL-6 and HMGB1 levels in the serum and artery conduit tissues were significantly higher in patients with CAD, and a positive correlation between IL-6 and HMGB1 was observed in CAD patients, suggesting that IL-6 is the key effector cytokine for TLR4-driven VSMC migration. This notion was further supported by direct evidence that neutralizing IL-6 antibodies abrogate TLR4-mediated VSMC migration (Figure 3). Additionally, CM from LPS-treated VSMCs significantly promoted VSMC migration and actin reorganization (Figure 2D–F and Figure 6). Taken together, these results suggest that TLR4 modulates vascular disease progression via VSMC migration, which might be mediated through an autocrine and/or paracrine actions of secreted IL-6.

VSMC migration contributes significantly to occlusive vascular disease. Cellular migration requires a coordinated polymerization of actin filaments. Many cytokines, growth factors, and mediators can function as chemoattractants for VSMCs. Indeed, we found that IL-6 elicited by LPS functions as a chemoattractant to induce VSMC migration (Figure 3 and Figure 6). We further demonstrated that LPS, IL-6, and LPS-CM-mediated VSMC migraton is through an actin reorganization-dependent mechanism. It has been reported that Rac1 activation plays a vital role in modulating cell migration, actin-based cytoskeletal rearrangements, and lamellipodia formation [39,40]. Consistent with this concept, we found that inhibition of Rac1 activity by pharmacological inhibitors diminishes LPS-induced VSMC actin cytoskeletal rearrangement and migratory activity (Figure 6), implicating a critical role of Rac1 in TLR4-mediated migration.

CREB, a widely expressed nuclear transcription factor, is considered to play an important role in gene regulation such as IL-6 gene expression. Much evidence indicates that several types of serine/threonine kinases are capable of phosphorylating CREB-1 on serine-133 to confer its transactivation activity [15,17]. Signaling pathway analyses reveal that p38 MAPK and ERK1/2 are involved in TLR4 agonist-induced CREB activation. Supporting our findings, it has been shown that p38 MAPK, ERK1/2, and JNK1/2 signaling is required for arachidonic acid-stimulated CREB phosphorylation or 15(*S*)-hydroxyeicosatetraenoic acid-induced CREB activation [16,19]. Furthermore, CREB-mediated IL-6 production is essential for TLR4-induced VSMC migration. In addition, p-CREB levels in artery conduit tissues were detectable in patients with CAD and positively correlated with IL-6 levels in the serum and artery conduit tissues. Taken together, these results suggest that TLR4 promotes VSMC migration by inducing IL-6 production, which is signaled via p38 MAPK- and ERK1/2-dependent CREB activation.

In summary, our findings indicate that TLR4 may play a pivotal role in VSMC migration, which contributes to atherosclerosis progression. We have provided novel evidence indicating that TLR4 promotes VSMC migration through CREB-triggered IL-6 production signaled via p38 MAPK and ERK1/2, which are mediated by MyD88- and TRIF-dependent pathways. These findings provide mechanistic insights into the functional importance of TLR4 in atherosclerosis and might be valuable for developing new therapeutic strategies against vascular diseases.

## 4. Materials and Methods

### 4.1. Materials

TLR2 ligand Pam3CSK4, TLR3 ligand Poly(I:C), TLR4 ligand LPS, TLR7/8 ligand CL087, TLR9 ligand CpG ODN, p38 inhibitor SB2021980, and ERK1/2 inhibitor U0126 were purchased from InvivoGen (San Diego, CA, USA). High-mobility group box 1 protein (HMGB1) was from Sigma-Aldrich (St. Louis, MO, USA). Kinases inhibitors including SB203580, PD98059, SP600125, and LY294002 were purchased from Calbiochem (San Diego, CA, USA); and antibodies against p38, phospho-p38, ERK1/2, phospho-ERK1/2, Akt, phospho-Akt (Ser473), JNK1/2, phospho-JNK1/2, CREB, phospho-CREB, NF-κB, and phospho-NF-κB were obtained from Cell Signaling (Danvers, MA, USA).

### 4.2. Primary VSMC Isolation and Treatment

Primary VSMC**s** were isolated from C57BL/6J mouse aortas and cultured in DMEM containing 10% FBS as previously described [20]. Briefly, mouse aortas were isolated from 18.5-day embryos and cut into small pieces that were digested using collagenase and elastase at 37 °C with mild shaking for 15 min. The enzymatic digestion was stopped by adding DMEM containing 20% FBS, and then the tissue explants were subjected to centrifugation. The tissue pellets were resuspended in DMEM containing 10% FBS and then placed into fibronectin-coated dishes. The primary VSMCs were passaged every 3–5 days, and all the experiments were performed with VSMCs at passage 6–8 from primary culture.

The VSMCs were typically subjected to serum starvation (0.5% FBS in DMEM) for 24 h and then pre-treated with either vehicle (0.1% DMSO) or kinases inhibitors for 30 min before stimulation with Pam3CSK4 (1 μg/mL) or LPS (100 ng/mL) or HMGB1 (50 μg/mL), unless otherwise specified. The period of treatments with TLR4 agonists varied depending on the experiments.

### 4.3. Cytokine Measurement

Cytokine levels in the culture medium were determined by a specific sandwich ELISA (Biosource, Camarillo, CA, USA) in 96-well microtiter plates coated with specific cytokine antibodies as previously described [20,41].

### 4.4. Western Blot Analysis

Analysis of endogenous protein levels by Western blotting was performed as previously described [42]. In brief, whole-cell lysates were isolated from VSMCs using RIPA buffer containing protease inhibitors. The cellular proteins were electrophoretically separated on 5% to 20% SDS-PAGE and immunoblotted with the desired specific antibodies.

### 4.5. Migration Assays

To assess migration, serum-starved VSMCs were plated onto the upper chamber of 24-well transwell plates (8-μm pore size; Corning Costar, Corning, NY, USA) in triplicate and treated with TE buffer, LPS or HMGB1 for 24 h. The bottom chambers were filled with 0.5% FBS medium containing either TE buffer or PDGF-BB (10 ng/mL) (Peprotech, Rehovot, Israel) as a chemoattractant. Cells migrating to the underside of the membrane after 4 h were quantified and normalized to the cell number of vehicle treatment.

In some experiments, 2 × 10^4^ quiesced VSMCs were placed in each well of the upper chamber and conditioned medium from VSMC treated with or without LPS was added to the bottom chamber as a chemoattractant in the transwell migration assays. After 24 h stimulation, VSMCs migrating through the filters were quantified.

To differentiate between chemotaxis and chemokinesis, checkerboard analysis was performed by plating conditioned medium from VSMCs treated with TE buffer or LPS either onto the upper or bottom chambers of the transwell plates. Cells migrating to the underside of the membrane after 24 h were counted and normalized to the cell number of VSMCs treated with vehicle.

### 4.6. Cell Viability Assay

The 3-(4,5-dimethylthiazol-2-yl)-2,5-diphenyl tetrazolium bromide (MTT) assay was used to measure cell viability in terms of metabolic turnover, as indicated by the mitochondrial reduction of MTT to purple formazan [43]. Briefly, after removing the supernatant by washing, the cells were incubated with MTT (0.5 mg/mL) for 1 h at 37 °C and solubilized in DMSO. The extent of formazan production was determined by using a microplate reader (Molecular Devices, Sunnyvale, CA, USA) at 540 nm.

### 4.7. siRNA Transfection

siRNA were purchase from Invitrogen (Carlsbad, CA, USA). The following siRNA sequences targeting mouse CREB were used: siRNA1 sense 5′-ACC AAA CUA GCA GUG GGC A-3′ and siRNA2 sense 5′-GAG GUC CGU CUA AUG AAG A-3′. The negative control sequence was 5′-UUC UCC GAA CGU GUC ACG UUU-3′. Transfection of VSMCs with scrambled control RNA or CREB siRNA was performed by RNAi Max (Invitrogen) or Gene Pulser Xcell Electroporation system (Bio-Rad, Hercules, CA, USA) according to the manufacturer’s instructions. In the electroporation system, 1 × 10^6^ VSMCs were mixed with 200 nM of siRNA in 100 μL of siRNA electroporation buffer in a cuvette. The VSMCs were electroporated with a 200 V. After 15 min incubation at room temperature, Electroporated VSMCs were placed in six-well plates and immediately incubated in a complete medium for 24 h before further treatment.

VSMCs were transfected with indicated siRNA using RNAi Max according to the manufacturer’s instructions (Invitrogen, Carlsbad, CA, USA). siRNA-transfected VSMCs were used for further treatment after 24 transfection.

### 4.8. F-Actin Staining

VSMCs were fixed with 1.5% paraformaldehyde in PBS for 5 min at room temperature and washed extensively. The cells then were blocked with Image-iT FX signal enhancer (Invitrogen) at room temperature for 30 min, permeabilized with 0.4% triton X-100 for 15 min at room temperature. After washing with PBS 3 times, the cells were stained with rhodamine phalloidin at room temperature for 1 h and visualized under a N-SIM SuperResolution microscope. The fluorescence intensities were quantified by NIS Elements Revolutionizes Imaging software (Nikon Corporation, Shinagawa-ku, Tokyo, Japan).

### 4.9. Patient Enrollment and Aortic Tissue Collection

Between September 2013 and July 2015, patients aged 20–90 years who underwent coronary angiography at the Tri-Service General Hospital were recruited for the study. Indications for coronary angiography included ischemic electrocardiography changes in treadmill exercise test, perfusion defect results in thallium scans or presentation of acute coronary syndrome. The exclusion criteria included inability to provide informed consent, presence of active infection, malignancy or autoimmune disorders, or pregnancy. A total of 227 patients with coronary artery disease (CAD) (161 men and 66 women; mean age, 61.2 ± 12.13 years) and 80 healthy control subjects (43 men and 37 women; mean age, 40 ± 13.34 years) without known systemic disease were enrolled in this study. The presence of CAD was confirmed by coronary angiography and CAD was defined as more than 50% angiographic diameter stenosis in one or more coronary arteries. Blood was drawn from patients prior to angiography and centrifuged at 2000× *g* for 15 min at 4 °C, aliquoted and stored in a −80 °C freezer until analysis. Serum levels of interleukin 6 (IL-6) and HMGB1 were determined using ELISA kits (R & D Systems, Minneapolis, MN, USA). Aortic specimens were collected from proximal ascending aortic punches made for aortocoronary anastomoses in 20 CAD patients (16 men and 4 women; mean age, 66.7 ± 12.13 years) who underwent coronary artery bypass graft surgery (CABG). The aortic punches were immediately snap-frozen in liquid nitrogen and stored at −80 °C for protein extraction. This study was conducted in accordance with the Declaration of Helsinki and was approved by the Ethics Committee on Human Studies at Tri-Service General Hospital, National Defense Medical Center in Taiwan (TSGHIRB No.: 2-102-05-104, Nov 2013 and 2-102-05-105, October 2013), and written informed consent was obtained from all the participants.

### 4.10. Statistical Analysis

Statistical analyses were performed using Graphpad Prism version 5 Software (GraphPad Software Inc., San Diego, CA, USA). All values were given as means ± S.D. The *t*-test was used to determine the statistical significance of the difference between the treatment and control groups, while one-way ANOVA was used to analyze multiple groups. Spearman’s correlation was used to analyze the clinical association between two variables. *p* Values < 0.05 were considered statistically significant.
